# Cortical Grey matter volume depletion links to neurological sequelae in post COVID-19 “long haulers”

**DOI:** 10.1186/s12883-023-03049-1

**Published:** 2023-01-17

**Authors:** Ted L. Rothstein

**Affiliations:** Department of Neurology, George Washington University, Washington, DC USA

**Keywords:** COVID-19, SARS-CoV-2, PASC, Long haulers, Voxel-based morphometry, Neurologic sequelae of COVID-19, Brain fog, Cognitive impairment

## Abstract

**Objective:**

COVID-19 (SARS-CoV-2) has been associated with neurological sequelae even in those patients with mild respiratory symptoms. Patients experiencing cognitive symptoms such as “brain fog” and other neurologic sequelae for 8 or more weeks define “long haulers”. There is limited information regarding damage to grey matter (GM) structures occurring in COVID-19 “long haulers”. Advanced imaging techniques can quantify brain volume depletions related to COVID-19 infection which is important as conventional Brain MRI often fails to identify disease correlates. 3-dimensional voxel-based morphometry (3D VBM) analyzes, segments and quantifies key brain volumes allowing comparisons between COVID-19 “long haulers” and normative data drawn from healthy controls, with values based on percentages of intracranial volume.

**Methods:**

This is a retrospective single center study which analyzed 24 consecutive COVID-19 infected patients with long term neurologic symptoms. Each patient underwent Brain MRI with 3D VBM at median time of 85 days following laboratory confirmation. All patients had relatively mild respiratory symptoms not requiring oxygen supplementation, hospitalization, or assisted ventilation. 3D VBM was obtained for whole brain and forebrain parenchyma, cortical grey matter (CGM), hippocampus, and thalamus.

**Results:**

The results demonstrate a statistically significant depletion of CGM volume in 24 COVID-19 infected patients. Reduced CGM volume likely influences their long term neurological sequelae and may impair post COVID-19 patient’s quality of life and productivity.

**Conclusion:**

This study contributes to understanding effects of COVID-19 infection on patient’s neurocognitive and neurological function, with potential for producing serious long term personal and economic consequences, and ongoing challenges to public health systems.

## Introduction

There is extensive literature on endemicity, clinical symptomatology, immunology and proposed pathogenetic mechanisms that underlie neurologic disorders among patients afflicted with COVID-19 (SARS-CoV-2) infections [1–4]. What appears lacking is an in depth understanding of possible structural brain changes that could underlie complex neurologic symptoms persisting in patients infected with COVID-19, and which may influence their quality of life and productivity [1, 3, 4]. COVID-19 infection can lead to prolonged systemic and neurological symptoms which may last many weeks or longer. The World Health Organization (WHO) has defined post COVID-19 infection at a minimum duration of 2 months [5]. Patients so afflicted are commonly referred to as “long haulers”, “long COVID,” or post-acute sequelae of COVID (PASC) [6]. Pooled prevalence data reveals the most frequently reported long term symptoms are chronic fatigue, dyspnea, myalgia, anosmia, ageusia, headache, and diarrhea [4, 7–9]. Other common symptoms which may have a major bearing on a patient’s quality of life involve cognitive impairment, memory loss, anxiety and sleep impairment [4, 7–9]. As reported in a meta-analysis of 1458 articles involving some 11,324 patients with long COVID symptoms, the prevalence of “brain fog” and memory issues were 32 and 27% respectively [2]. “Brain fog” is not a recognized medical term but refers to a reduction in alertness or mental acuity, impaired ability to concentrate, confusion, or “clouding of consciousness” [1, 9].

The prevalence of Neurologic and Psychiatric morbidity has been detailed in a retrospective study in which electronic health records of 236,379 patients were reviewed [10]. One third of those patients surviving COVID-19 had substantial neurological and psychiatric morbidity 6 months following their infection [10]. A Danish study of 445 non-hospitalized COVID-19 patients found persistent symptoms- mainly fatigue and difficulties with memory and concentration- occurred in 36% of symptomatic cases when followed > 4 weeks [11]. A meta-analysis of 43 studies assessed 12 or more weeks following COVID-19 diagnosis disclosed that 22% exhibited cognitive impairments resulting in substantial functional impairment [6]. A comprehensive review of the US Department of Health database was performed by Xu et al. and estimated that the burden of neurologic disorders 1 year following COVID-19 infection was 42% [12]. Identical prevalence values for neurologic sequelae were found in a study of 509 COVID-19 patients among those hospitalized at Northwestern University [4]. Neurologic syndromes in the acute phase have included encephalopathy, encephalitis, macro/microhemorrhages, central venous thrombosis, acute disseminated encephalomyelitis (ADEM), myelitis, Guillain-Barré syndrome, Miller Fisher variant, persistent fatigue, insomnia, seizures and neuromuscular disorders [4, 12–15].

Graham et al. reviewed neurologic symptoms among their first 100 non-hospitalized long COVID patients and found that patients were mainly younger and mostly female [1, 4]. In subsequent follow up studies, there were no major changes in the frequency of most of their neurologic symptoms at a median of 14.8 months or 2 years after disease onset [1, 4, 16]. The risk of death and multiple post infectious sequelae can still occur in vaccinated patients, although vaccination confers partial protection at slightly lower risk [17]. However, the range of post-acute symptomatology is no different among those with prior vaccination when compared to unvaccinated individuals [17].

The purpose of the current study is to quantify volumes of whole and forebrain parenchyma, as well as key GM structures important for cognition, memory, and other neurologic functions in “long haul” patients following COVID-19 infection. Results are based upon findings obtained with Brain MRI supplemented with NeuroQuant® 3D VBM, among a cohort of 24 consecutive post COVID-19 infected long-term patients experiencing neurologic sequelae. Each result has been compared with sex and age matched healthy controls drawn from an existing normative database provided by the developer of NeuroQuant® (Cortechs Laboratories, San Diego, California) with values based on their percentage of intracranial volume.

## Methods

The current study analyzed 24 long COVID patients who were evaluated at a median of 85 days after laboratory confirmation of COVID-19 infection. Each patient met Infectious Diseases Society of America serologic criteria for COVID-19 infection [18]. Serologic testing was performed at the George Washington University Pathology Laboratory using Cepheid Xpert Xpres SARS-CoV-2 real time polymerase chain reaction (PCR) test for qualitative detection of COVID-19 coronavirus. Neuroimaging employed 3D VBM NeuroQuant® software (NeuroQuant® v2.3), an automated software package which acquires, segments, and quantifies unenhanced 3-dimensional T1 weighted volumetric images of multiple brain structures not apparent with conventional MRI [19–26]. NeuroQuant® was performed following acquisition of conventional MRI upon Siemens 3 T Skyra scanners each with a 16-channel head coil (Siemens Medical Systems, Erlangen, Germany). Quantitative MR applications using NeuroQuant® 3D VBM have allowed for precise measurements of GM tissue damage which can lead to cognitive and neurologic disability accumulation [19–26]. Automated 3D VBM techniques have been shown to perform at least as well as, or better than manual segmentation performed by expert Neuroradiologists, Radiologists and Neurologists, who have specialized training and expertise in anatomic labeling of MR images [24–26].

NeuroQuant® software uses only 3D sagittal T1 sequences for volume measurement and quantifies various volumes of brain structures, comparing them against a normative data base adjusted for age, sex, and intracranial volume. No other sequences were included for NeuroQuant® analysis in the present study. The acquisition parameters of the neuroimaging sequence is as follows: TR/RE/TI = 2300/2.98/900, Flip Angle = 9, BW =240 Hz /Px, 240 × 256 matrix. 160 slices, voxel size + 1 × 1 × 1.2 mm/ scan time 9:14. Automated segmentation method used by NeuroQuant® are evolved from semi-automated methods relying on probabilistic atlas-based methods and provide volumetric analysis of each segmental structure [19–25]. The segmentation procedure assigns a neuroanatomic label to each voxel on the basis of probabilistic information automatically that maximizes the probability of input given previous probabilities derived from atlases. The software deletes non-brain tissue using active contour models and separates a number of anatomic structures using the same probabilistic atlas. NeuroQuant® MRI automated technique quantifies whole brain and forebrain parenchyma, CGM and deep GM nuclei volumes including measurements of hippocampus, and thalamus for which age and sex based normative data is available. The output of NeuroQuant® computer-automated analysis includes a report with volumetric information and a set of DICOM-formatted brain images. Calculations of total brain and forebrain volumes, and derived percentiles of intracranial volumes of CGM, hippocampus and thalamus for each sample is based upon a NeuroQuant® normative database, which is not made available to its users. Additional details of NeuroQuant® methodology have previously been described elsewhere [27].

### Statistical analysis

Details of each study subject’s self-reporting of neurological complaints are provided. The means and standard deviations from the NeuroQuant®-based percentiles for each brain region are reported. For each region there was conducted a 1-sample sign test of the null hypothesis that the median for that brain region is equal to the median for the normative database drawn from healthy controls. Since percentiles are adjusted for age, sex, and intracranial volume by the NeuroQuant® database, this means that the percentiles of individuals in the study are comparable – regardless of age, sex, and intracranial volume. Hence, we are able to use simple 1-sample sign test *p*-values from each sign test as reported. A *p*-value less than 0.01 is considered statistically significant. Only volumes of CGM and thalamus as a percentile of intracranial volume reach the level of statistical significance in this study.

## Results

All patients were characterized as “long haulers” as they experienced neurologic symptoms lasting at a minimum of 8 weeks [1, 4, 5], according to criteria established by WHO and NICE guidelines [5, 28]. They had relatively mild respiratory symptoms which brought them to George Washington University Outpatient Clinic or Hospital for COVID-19 testing, and none required oxygen therapy, hospitalization or assisted ventilation. Each patient was interviewed by Telehealth video linkage, and self-reported neurological complaints including details of their cognitive ability, and other neurologic symptoms, as well as the degree to which their symptoms were affecting their quality of life. Their average age was 46.9 years (range 22–60 years), 19 were female (79%), and each had no prior record of neurologic impairments, cognitive decline or chronic fatigue. Patients over 60 years of age were excluded from analysis to avoid GM changes that could be associated with aging.

Patients self-reported an average of 4 neurological complaints, the most common being cognitive impairment, which could be described as poor memory or concentration, mental confusion or “brain fog” (91.6%), followed by fatigue (87.5%), new onset headache (41.6%), word finding or speech impairment (41.6%), altered sense of smell or taste (29.1%), myalgia (16.7%), paresthesia (16.7%), new anxiety or depression (16.7%), sleep disorder (12.5%), and dizziness or vertigo (12.5%). Pre-existing comorbidities included depression or anxiety (29.1%), headache (16.7%), type 2 diabetes mellitus (12.5%), and seizure disorder (4.1%).

On standard Brain MRI using conventional 3 Tesla (3 T) Siemens scanners, one patient had a small pituitary microadenoma, while 3 of the older study patients had nonspecific white matter changes attributed to “microvascular ischemic change”. Microvascular ischemic changes are commonly seen due to aging and are not associated with a particular symptomatic profile. None of the remaining 20 study patients had structural abnormalities on standard Brain 3 T MRI.

Table 1 provides patient characteristics of 24 post COVID-19 “long haulers” including their sex, age, and presenting neurologic symptoms. NeuroQuant® 3D VBM analyzes brain volumes of whole brain and forebrain parenchyma, hippocampus, thalamus, and CGM based on their intracranial percentiles. Median volumes for CGM and thalamus were found to be different from adjusted values derived from the NeuroQuant® normative database when compared to age and sex matched healthy controls and achieved statistical significance.

**Table 1:** Patient Characteristics with NeuroQuant processing evaluates brain volumes as intracranial percentiles compared to normal controls

*Patient* *Age/Sex*	*Whole* *Brain*	*Forebrain Parenchyma*	*CorticalGrey* *Matter*	*Hip po* *campus*	*Thal amus*	*Presenting Neurologic Symptoms*
1.22 M	79	91	35	57	92	Anosmia, ageusia, brain fog, memory loss, fatigue, headache, impaired concentration, sleep disorder
2.29 F	55	49	15	87	92	Brain fog, memory impairment, inability to focus or concentrate, fatigue, hypersomnolence, word finding difficulty, headache
3.31 F	33	33	38	89	99	Brain fog, memory loss, forgets appointments, fatigue
4.34 F	97	96	35	98	99	Can’t complete tasks, forgets appointments, memory loss, fatigue
5.40 F	22	5	11	37	94	Anosmia, ageusia, memory loss, stuttering speech, fatigue
6.42 M	80	62	16	91	99	Anosmia, ageusia, fatigue, memory loss
7.46 F	21	31	3	94	94	Brain fog, memory loss, fatigue, forgot her age and home address, stuttering speech
8.46 F	42	52	13	99	65	Fatigue, emotional lability, difficulty with reading and comprehension
9.46 F	93	84	61	87	75	Fatigue, headache, impaired memory and word finding, emotional lability
10.46 F	86	63	4	99	99	Brain fog, memory loss, fatigue, impaired comprehension, can’t organize thoughts, word finding impairment, headache
11.46 F	28	19	25	99	60	Memory loss, forgetful, fails to pay bills, sleep disorder, depression
12.47 F	10	6	40	94	35	Fatigue, olfactory hallucinations, memory loss, headache, depression
13.47 F	38	47	15	99	99	Brain fog, memory loss, fatigue, headache, poor concentration
14.48 F	20	10	10	37	94	Anosmia, ageusia, memory loss, stuttering speech, fatigue
15.49 F	69	44	29	49	99	Memory impairment, forgetful, repeats herself, fatigue, microadenoma
16.56 F	81	80	61	87	75	Anosmia, ageusia, gets lost in familiar places, can’t follow instructions
17.56 F	73	63	21	98	92	Memory loss, forgets conversations, can’t recall what she has read, repeats self
18.56 M	29	31	13	89	92	Fatigue, memory loss, inability to concentrate, headache
19.56 F	67	69	33	99	57	Brain fog, impaired memory, fatigue, hypersomnolence
20.58 F	10	6	40	97	59	Fatigue, olfactory hallucinations, memory loss, headache, depression
21.58 F	58	27	14	99	98	Brain fog, memory loss, fatigue, hypersomnolence
22.60 M	13	13	14	36	99	Anosmia, ageusia, memory loss, stuttering speech
23.60 F	19	21	20	98	72	Math teacher can’t perform simple math, memory loss, fatigue
24.60 M	59	39	2	14	84	Forgetful, directionally impaired, can’t locate items in his kitchen, fatigue

**Fig. 1:**
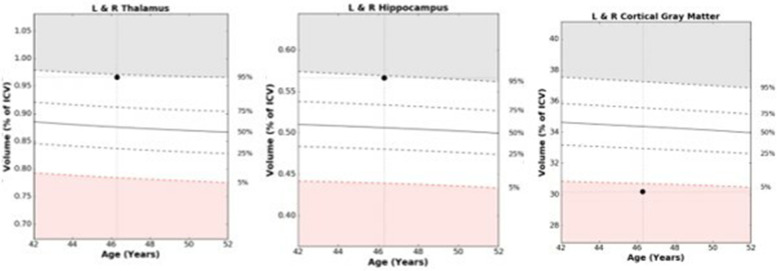
An example of NeuroQuant® numeric data acquisition and 3-dimensional voxel-based morphometry results in patient #7 is provided in Fig. 1 which tracks volumes of thalamus, hippocampus and cortical grey matter as their percentage of total intracranial volume (ICV) when compared with normative database drawn from healthy controls and adjusted for age, and sex. Abnormal range is defined as beyond the white zone at the 5th percentile or less, or 95th percentile or more, at a given age

Table 2 Provides the mean (sd) of the percentiles for each brain region sampled from the cohort of 24 study COVID-19 patients when compared to the NeuroQuant® normative database provided by Cortechs Laboratories. The *p*-value column provides the *p*-value for the 1-sample sign test. The *p*-values for CGM and thalamus are both < 0.01, indicating that the median volumes for these regions reveal a statistically significant difference from the adjusted values.

**Table 2:** Statistical Analysis for 24 COVID-19 infected “long haulers”

Brain Region	Mean (sd)	***p***-value
Whole Brain	48.41 (29.15)	1.0000
Forebrain	41.05 (27.27)	0.2863
Cortical Grey Matter	24.27 (17.83)	0.0009
Hippocampus	80.77 (26.57)	0.0169
Thalamus	83.27 (18.28)	< 0.0001

Figure 1 displays structural images processed through NeuroQuant® automated sequestration software to provide comparisons of a given individual's structural brain volume measurements when compared to a normative database of age and sex matched healthy controls. This technique provides operator independent quantitative measures of key brain regions including thalamus, hippocampus and CGM.

Further review of results of study patients when compared with the NeuroQuant® dataset indicates that while CGM volumes are diminished, thalamus and hippocampal volumes are larger than expected, compared with samples from the normative database of sex and age matched healthy controls. There is no evidence that median volumes for whole brain or forebrain parenchyma differ from median volumes for those regions in the normative database.

The findings in our study of 24 COVID-19 infected patients indicate a more global deficiency of CGM volume than has been previously recognized. One explanation for these results is that while total brain volume remains stable, CGM volume is depleted, and relative intracranial volumes of thalamus and hippocampus appear enlarged as their percentiles are computed and compared using normalized volumes. Alternatively, some studies have demonstrated that post-traumatic stress, which could be brought about by the consequences of COVID-19 infection, can induce greater hippocampal and basal ganglia volumes as a functional response to coping with increased physical and mental stress [29]. There is also a possible contribution from macroscopic volume changes associated with adult hippocampal neurogenesis [29, 30].

## Discussion

There remains a considerable gap in our understanding of how COVID-19 produces damage to the central nervous system (CNS) [1, 3, 4, 14], which has resulted in much speculation upon whether and how COVID-19 might affect neurons in the brain [3, 4, 31]. The current study analyzed 24 post COVID-19 infected long-term patients who underwent Brain MRI with NeuroQuant® 3D VBM analysis and were found to have a statistically significant deficiency of CGM volume as a possible contributing factor to their neurological decline. It has long been recognized that large cortical lesions or injuries damage cognitive and other brain functions roughly in proportion to the extent of amount of tissue lost [32]. CGM is responsible for a wide range of behaviors including memory, cognition, language, perception, volitional movement, and emotions [33]. However, it is not known whether abnormal findings in the study patients could have predated their COVID-19 infection, although this would have been unexpected as they lacked cognitive issues or chronic fatigue prior to their COVID-19 exposure.

Identifying abnormalities in GM volumes based on imaging studies could help clinicians and patients understand the source for neurological sequelae in long COVID-19 survivors.

There are a few reports which assess GM volumes obtained in COVID-19 patients, and those have produced variable and inconsistent results.

A UK Biobank study involved 401 participants receiving conventional MRI twice, both prior to and following COVID-19 infection, with an average gap of 38 months, which were compared to a parallel group of 384 uninfected controls [34]. There were greater reductions in parahippocampal gyrus and orbitofrontal cortex, as well as whole brain size in infected patients, which was attributed to their infection [34]. Cognitive testing revealed a greater decline in executive function among their infected cohort [34].

Automated data driven analysis can quantify changes in brain structure and tend to be more reproducible when compared with more conventional MR imaging [35]. Preliminary study of 54 patients with severe COVID-19 infections, using automated 3D VBM measurements of brain tissue volumes, found volume loss in frontal, temporal, and bilateral mesiotemporal GM, compared with asymptomatic COVID-19 patients and healthy controls [8]. A further report identified decreases in cortical thickness and subcortical volumes mainly in the left frontal and limbic systems in patients with both mild and severe infections, despite having no neurologic manifestations during their acute phase [36]. Sanabria–Diaz and colleagues describe findings in 33 selected patients with different levels of post COVID-19 infection severity and identified lower cortical volumes in orbitofrontal and cingulate regions than in controls [37]. In addition, cortical reconstruction using Freesurfer 3D morphometry showed lower GM volumes in frontal, limbic, parietal and temporal brain regions [37]. Duan et al. analyzed a series of 58 acutely ill COVID patients using Computer Tomographic (CT) source-based morphometry (SBM) to assess GM changes as compared to 62 non-COVID controls [35]. Lower GM volumes were detected in frontal gyri which were associated with increased levels of disability, which occurred in their patients receiving oxygen therapy, and in temporal lobes in those with fevers [35]. Interestingly, no significant GM volume changes occurred in patients infected with COVID-19 compared to those without COVID infection in any brain region [35]. However, this study having been based upon CT images might be less reliable as compared to those in which MRI 3D VBM was used to assess alterations induced by COVID-19. By contrast, Lu et al., described 60 recovered COVID-19 long-term patients having an increase in GM volumes after 3 months in bilateral hippocampi, insulas, left Rolandic operculum, left Heschl’s gyrus, and right cingulate gyrus, when compared to controls [38]. These changes appeared to correlate with memory impairment and ageusia. The authors speculated that connecting white matter fibers were disrupted due to a massive “cytokine storm” acting as a channel for intracranial viral transmission [38]. Tu and colleagues analyzed brain structure volumes and functionality in 126 COVID-19 survivors at 3 months, and 47 survivors at 6 months, after being discharged from hospital in Wuhan, China, and were experiencing varying levels of post-traumatic stress disorder. They identified significant volume enlargements in bilateral hippocampus and amygdala which they proposed was due to functional compensation in coping with the ongoing stress of COVID-19 infection [39]. Those who survived mild infection had earlier onset of cognitive decline [39]. Besteher et al. using 3D VBM found significantly enlarged GM volumes in long-COVID patients involving hippocampus, thalamus, fronto-temporal areas, insula, amygdala, and basal ganglia in both hemispheres in a series of 30 patients with neuropsychiatric disorders when compared to controls [40]. Additionally, a study of 46 COVID-19 infected patients with chronic fatigue who were scanned with MRI 2 weeks after hospital discharge (and after converting to COVID PCR negative), were found to have higher GM volumes within the basal ganglia (putamen, pallidum) and limbic system (bilateral hippocampus, parahippocampal gyrus, left amygdala, insulas, and right entorhinal area) [41]. Hippocampal GM enlargement was also found in patients with long term memory deficits following the occurrence of transient global amnesia (TGA) which was attributed to adult hippocampal neurogenesis and macroscopic changes [29, 30]. Paradoxically, larger GM volumes in the hippocampus were associated with poorer performance upon global cognition testing [29].

 GM changes in long COVID have also been documented using ^18^fluorodeoxyglucose Positron Emission Tomography (PET) with decreases in metabolic activity among 28 consecutive outpatients, identified mainly in the right frontal and temporal lobes, but including orbito-frontal cortex and internal temporal and frontoparietal lobes [42]. Other studies involve decreases in cingulate cortex [43], or bilateral rectus/orbital gyrus including the olfactory gyrus, pre and post central, temporal and fusiform gyri, which consistently enabled infected patients to be distinguished from healthy controls [44]. Those patients with clusters of hypometabolism experienced hyposmia/anosmia, cognitive impairment, chronic pain and insomnia [44]. In a systematic review of PET studies in COVID-19 long-term patients, results were inconsistent in those complaining of fatigue and memory loss, as some had extensive areas of hypometabolism in limbic and subcortical structures, while others were found to have no metabolic abnormalities [45]. Further, no changes of regional cerebral glucose metabolism were found among 14 long COVID patients reporting neurologic symptoms sufficient to hamper their ability to work [46].

While additional data from standard Brain MRI may be relevant as contributing to understanding long COVID neurological symptoms, results so far have been inconsistent and inconclusive. Most standard imaging studies of COVID-19 to date are reports of single cases or limited case series which reveal heterogeneous brain involvement with widespread white matter (WM) hyperintensities, hypoperfusion and cerebrovascular damage [47]. Upon review and meta-analysis of neuroimaging findings in 1394 COVID-19 patients from 17 studies, the major findings were olfactory bulb abnormalities (23.1%) and alterations in WM (17.6%) [48]. Another MRI investigation described imaging results in 37 of 190 consecutive patients with severe COVID-19 infection, which disclosed a wide spectrum of abnormalities on fluid attenuated inversion recovery imaging (FLAIR) including medial temporal lobe atrophy, confluent WM hyperintensities, and extensive or isolated WM microhemorrhages [49]. In a study of 242 patients who had head CT or Brain MRI performed within 14 days of COVID-19 diagnosis, the most common finding was non-specific WM microangiopathy (55.4%), followed by chronic infarction (19.4%), acute or subacute infarcts (5.4%), and intracranial hemorrhage (4.5%) [50]. All those patients with cerebral infarcts and hemorrhages had focal neurologic findings on examination while none of the 102 patients described with “altered mental status” had these changes [50]. An additional study found that 23% of COVID-19 patients with neurologic signs or symptoms had varying image abnormalities of which the most common were scattered microhemorrhages (60%), followed by acute or subacute infarcts (25%), and WM hyperintensities (20%) [51]. Fitsiori et al. described patients with moderate or critical COVID-19 illness who had unusual patterns of microbleeds affecting corpus callosum, subcortical and parahippocampal regions, middle cerebellar peduncles, and internal capsules [52]. Another report described 13 patients with severe COVID-19 disease, of whom 11 were found to have leptomeningeal enhancement [53], but these findings were subsequently disputed [54]. High field images using an 11.7 Tesla scanner on postmortem brain tissue disclosed areas of microvascular injury, microhemorrhages and fibrinogen leakage [55]. Other reviews concluded there were no specific radiologic findings which were related to cognitive impairment following COVID-19 infection [3], or distinguishable from small vessel changes (microvascular ischemia) associated with aging [56].

There is limited knowledge about fundamental mechanisms of COVID-19 infection and possible interactions between viral and non-viral factors. This has resulted in much speculation on whether and how COVID-19 affects neurons in the brain [3, 14, 31]. Among proposed etiologies are immune-mediated processes that are induced by the infection, direct infiltration of the CNS, and virus-induced hyperinflammatory and/or hypercoagulable states inducing thrombosis [3, 14, 57, 58]. There are cases of acute COVID-19 patients developing ischemic stroke resulting from hypercoagulability, inflammation, cardiac dysfunction, and endothelial inflammation [13–15, 59, 60]. COVID-19 can infect cerebrovascular endothelium resulting from circulating antiphospholipid autoantibodies which induce cellular activation and dysfunction leading to thrombosis [61]. Another proposed mechanism is entry of the virus through the cribriform plate and olfactory bulb which would explain anosmia and ageusia that can develop acutely in some patients infected with COVID-19 [62]. Loss of taste and smell was found to be the result of T cell infiltration in the nasal olfactory epithelium on biopsied samples from hyposmic patients [63]. Pathologic changes have been detected in the olfactory bulbs of patients who died from their COVID-19 infection, and viral specific RNA has been extracted from their olfactory tracts [62]. Once the virus enters the systemic circulation, it could invade neural tissue by means of neurotropism related to the interaction of membrane-bound angiotensin converter 2 receptors (ACE-2) on vascular endothelium [64]. Astrocytes which help form the Blood Brain Barrier (BBB), and brain microvascular endothelial cells express ACE-2 receptors which can allow COVID-19 virus to bind and alter tight junctions leading to increased BBB permeability and neuroinflammation [65–67]. Indirect mechanisms related to “immune dysregulation” have also been cited as a putative source for brain damage by inducing a “cytokine storm” triggered by infection [21, 68, 69]. Cytokine storms are thought to be the result of immune system transition from the usual adaptive response to a disproportionate inflammatory response resulting in organ dysfunction, organ failure, and in extreme cases, can lead to death.

Histopathological study has not disclosed clusters of inflammatory cells surrounding infected cells in human brain, which would be more typical for viral encephalitis [70]. Moreover, a Swedish study of adults with COVID-19 infection and neurologic symptoms, when compared with healthy asymptomatic controls, found that the majority had viral nucleocapsid antigen in CSF, which correlates with CNS immune activation, but not with direct viral invasion [71]. In only 10 of 133 reported series focusing upon the diagnosis and treatment of patients diagnosed with immune-mediated symptoms of COVID-19, were specific neuronal or ganglioside antibodies identified [57]. Another putative mechanism utilized a newly established brain organoid model with innately developing microglia, which upon exposure to COVID-19 infection induced neuronal cell death due to microglial post synaptic terminal elimination [72].

 Mechanisms contributing to COVID-19 Neuropathology can be identified through postmortem studies. Reiken et al. performed autopsy studies on 10 post COVID-19 patients which revealed the accumulation of tau proteins and neurofibrillary tangles similar to neuropathology typical of Alzheimer’s disease [73]. Their laboratory demonstrated modifications of ryanodine receptors which control intracellular passage of calcium, and which in Alzheimer’s disease accumulates tau proteins and production of neurofibrillary tangles [73]. Another post-mortem study identified pathological changes of β-amyloid aggregation and plaque formation accompanying tauopathy, neuronal degradation and cell death [74]. Brain tissue analysis of single cells disclosed CD8 + T cell lymphocyte infiltration and microglial activation without detection of the COVID-19 virus, suggesting significant and persistent inflammation [75]. A further post-mortem study of 9 patients showed microvascular injury by immune complexes with leakage of serum proteins into brain parenchyma accompanied by widespread endothelial activation. These changes resulted in breakdown of the BBB, microthromboses, microglial nodules, perivascular inflammation, neuronal loss and neuronophagia, proposed as central to the development of neurologic manifestations [76]. An additional report described perivascular macrophage accumulation, microgliosis and microglial aggregates within the brains of COVID-19 patients studied postmortem [77]. Matschke et al. found 21 of 40 patient brains were positive for SARS-CoV-2 RNA or protein, and 16 of 40 were positive by nucleocapsid or spike protein immunohistochemistry [78]. Cytotoxic T lymphocytes and microglial activation were identified as present in cranial nerves, brainstem and cerebellar tissue [78]. However, the presence of SARS-CoV-2 in brain tissue did not correlate with the severity of neuropathologic changes [78]. In a post-mortem study of seven COVID-19 patients, microglial activation was present in brainstem, and olfactory bulb which was thought to represent a histopathological correlate of critical illness-related encephalopathy and not a disease-specific finding [79]. SARS-CoV-2 virus RNA has been found in other human brain tissue where it infects astrocytes, and to lesser extent neurons. The infection has been implicated through non-canonical mechanisms involving Spike-NRPI interactions, which can induce metabolic changes that deplete metabolites used to fuel neurons and synthesize neurotransmitters [69]. Therefore, infected astrocytes could impair neuronal viability which would give rise to structural changes that develop in the brains of COVID-19 patients [69].

## Conclusions

The strength of the present study is that it provides evidence for deficiencies limited to CGM volumes found in a cohort of 24 post COVID-19 “long haulers”. Depletion of CGM volume likely contributed to their neurologic sequelae and in particular, cognitive impairment and “brain fog”. These results underscore the potential of COVID-19 infection to produce neurocognitive effects with serious long term personal and economic consequences which could impair COVID-19 patient’s quality of life and productivity. There are also likely to be ongoing challenges to public health systems. A deeper understanding of the neuropathologic etiologies which underlie COVID-19 infection would potentially have implications for identifying measures which could mitigate their adverse effects. A larger investigation of post-COVID-19 patients utilizing Brain MRI with 3D VBM is necessary to confirm and expand the results of this study. Longitudinal clinical and 3D VBM imaging studies would be of value in determining the extent to which recovery of neurologic function, and restoration of depleted CGM volume is possible. Moreover, serial studies could identify potential neuroprotective therapies in post COVID-19 “long haulers”. In sum, this study contributes to understanding the underlying anatomic changes induced by COVID-19 infection and their effect on neurocognitive and neurological function, with the potential of producing serious long term personal and economic consequences, and challenges to public health systems.

### Limitations

This study has limitations beyond the small sample size. The patients in our series were heterogeneous as they were likely infected by different variants of COVID-19. There is no data on their vaccination status or treatments patients may have received during the acute or subsequent phases of their infection. The patient’s clinical data is limited to self-reported complaints with attention directed to signs and symptoms as defined by previous clinical studies, which can lead to reporting bias [80]. Moreover, patients were evaluated at George Washington University Cognitive Disorders Clinic which may have skewed the prevalence of cognitive impairment and “brain fog”. Comprehensive analysis could not be performed on the study patient’s clinical and cognitive status due to limitations imposed by the pandemic. Another limitation is the lack of measures to assess the degree of severity of each patient’s neurological symptoms. Further, there are no baseline MRI studies taken prior to the onset of long COVID to compare volumes of GM structures before and after their infection. Shortcomings have been identified in the process of segmentation of brain regional structures using automated techniques [81–85]. Several biologic confounders can influence the volumetric results such as daily fluctuations in brain volumes, comorbidities, aging, state of hydration and head size [81–85]. Future evaluations would benefit by including comprehensive computerized cognitive testing which preferably should be accessible from remote locations.

## Data Availability

The study includes reference articles which are available via PubMed. All information analyzed in this study was collected in a dataset at George Washington University and is available from the corresponding author on reasonable request.
